# Potential roles of inter-chromosomal interactions in cell fate determination

**DOI:** 10.3389/fcell.2024.1397807

**Published:** 2024-05-07

**Authors:** Junko Tomikawa

**Affiliations:** Department of Maternal-Fetal Biology, National Research Institute for Child Health and Development, Tokyo, Japan

**Keywords:** 3D nuclear organization, Hi-C, lncRNA, totipotent cells, epigenetics

## Abstract

Mammalian genomic DNA is packed in a small nucleus, and its folding and organization in the nucleus are critical for gene regulation and cell fate determination. In interphase, chromosomes are compartmentalized into certain nuclear spaces and territories that are considered incompatible with each other. The regulation of gene expression is influenced by the epigenetic characteristics of topologically associated domains and A/B compartments within chromosomes (intrachromosomal). Previously, interactions among chromosomes detected via chromosome conformation capture-based methods were considered noise or artificial errors. However, recent studies based on newly developed ligation-independent methods have shown that inter-chromosomal interactions play important roles in gene regulation. This review summarizes the recent understanding of spatial genomic organization in mammalian interphase nuclei and discusses the potential mechanisms that determine cell identity. In addition, this review highlights the potential role of inter-chromosomal interactions in early mouse development.

## Introduction

Development of the chromosome conformation capture (3C) assay, a proximity ligation assay used with PCR, was a breakthrough in chromatin biology (Dekker et al., 2002). Next-generation sequencing has led to the development of several 3C-derived approaches to assess contact frequencies between two genomic loci: circular 3C (4C)-seq to identify loci that interact with a single locus (Simonis et al., 2006; Wei et al., 2013) and high-throughput 3C (Hi-C) to map genome-wide interactions (Lieberman-Aiden et al., 2009; Duan et al., 2010). These methods marked an era of three-dimensional (3D) genome structural analysis of interphase nuclei. Recent developments in super-resolution microscopy and imaging techniques have facilitated high-throughput examination of chromatin conformation in single cells (Su et al., 2020; Takei et al., 2021). CRISPR/Cas9 is an adaptive immune system that cleaves exogenous gene elements in certain microorganisms. Endonuclease-deficient Cas9 facilitates targeted gene regulation through epigenetic editing and imaging of specific genomic loci in living cells. The combination of live imaging and CRISPR-based systems has improved the understanding of chromatin contact dynamics (Gu et al., 2018; Shaban and Seeber, 2020). Here, the recent advances in 3D genomics are summarized and future research directions are discussed.

## Principles of genomic structure and nuclear organization

The mammalian genome contains a large amount of DNA with a total length of 2 m. Folding and organizing genomic DNA in the interphase nucleus is critical for gene regulation and cell fate determination. Proximity ligation-based genome-wide approaches such as Hi-C have drastically enhanced our understanding of the association between transcriptionally active/inactive states and 3D structural DNA patterns in the mammalian genome.

Hi-C revealed that the genome is classified into two spatial compartments: “A” compartment containing actively transcribed genes and “B” compartment containing transcriptionally silent domains (Lieberman-Aiden et al., 2009; Rao et al., 2014). The A compartment, located inside the cell nucleus, is gene-rich, marked by histone modifications for active transcription, and possesses high GC content. In contrast, the B compartment, located near the nuclear periphery, is gene-poor, condensed, and marked by histone modifications for gene silencing. Initially, the A/B compartments were considered to be approximately 1–10 Mb in size and comprise many topologically associating domains (TADs) (Lieberman-Aiden et al., 2009; Dixon et al., 2012), but recent high-resolution analysis has revealed that most compartments are less than 100 kb in size. The median compartment size is 12.5 kb (Harris et al., 2023). TADs are considered to be formed by active extrusion of chromatin loops (Sanborn et al., 2015; Fudenberg et al., 2016). Loop extrusion factors such as cohesin bind to chromatin fibers and chromatin loops gradually expand until they either drop out, encounter each other, or encounter extrusion barriers that define the TAD boundary (Nuebler et al., 2018). TADs regulate transcriptional activation by restricting enhancers to target promoters within the same loop. Genes within the same TAD synchronize their expression patterns and are often co-regulated during cellular differentiation (Szabo et al., 2019). Disruption of TAD boundaries can result in the aberrant expression of genes that should not be expressed, leading to developmental abnormalities and diseases (Giorgio et al., 2015; Lupiáñez et al., 2015).

Although TAD structure was thought to be relatively constant among different cell types across species, later studies adopting high-resolution analyses have revealed that there are sub-TADs within TADs and that many of sub-TADs change their structures during cell differentiation (Dixon et al., 2015; Ibn-Salem et al., 2017; Oudelaar et al., 2020; Georgiades et al., 2023). A/B compartments are cell type-specific and can switch over during cell differentiation and lineage commitment (Lieberman-Aiden et al., 2009; Nora et al., 2017; Rao et al., 2017; Schwarzer et al., 2017; Miura et al., 2019).

Chromosomal DNA is compartmentalized into constant intranuclear spaces called “chromosome territories” (Cremer and Cremer, 2010). Nuclear locations of chromosome territories in human and mouse cells are not random (Croft et al., 1999; Cremer et al., 2003). Gene-rich loci were more internally localized, whereas gene-poor loci were more peripherally localized. The distribution of chromosome territories is tissue-specific (Parada et al., 2004; Bolzer et al., 2005) and the territories are lost during mitosis and recovered after mitosis (Gerlich et al., 2003). Genomic DNA folds in the nuclear space in an orderly manner at multiple levels via different mechanisms.

## Inter-chromosomal interactions revealed by proximity ligation-independent methods

**FIGURE 1:**
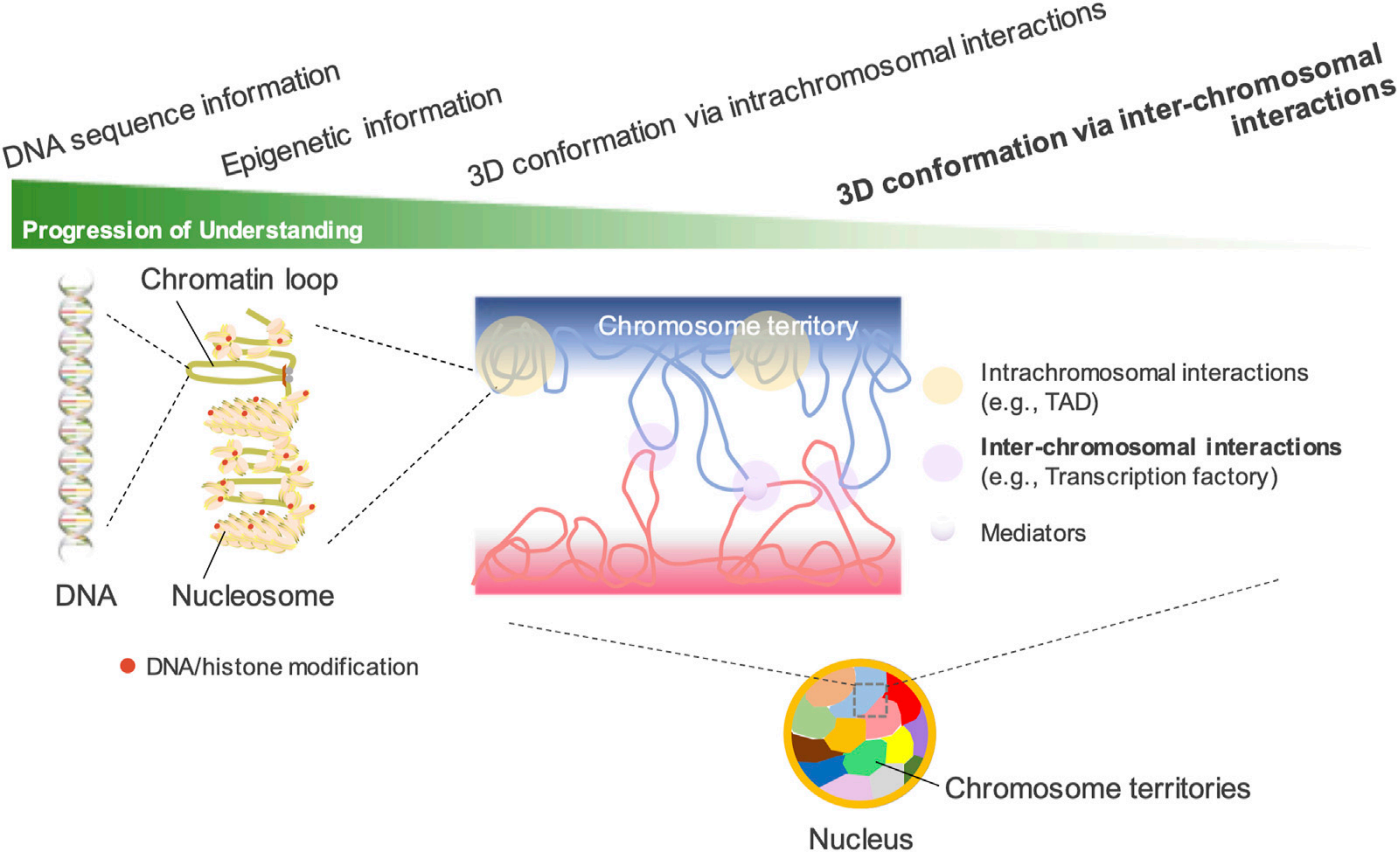
Understanding the spatial organization of the nuclear genome. In interphase nuclei, each chromosome occupies its own distinct territory, called “a chromosome territory.” In each territory, chromatin forms highly organized structures such as TADs and A/B compartments. Hi-C technology has greatly advanced the understanding of the 3D genomic structures configured by intrachromosomal interactions. However, the understanding of inter-chromosomal interactions has lagged far behind, as their presence can be detected by DNA fluorescence *in situ* hybridization but is difficult to detect by Hi-C. The existence of chromosome territories, which limit the interactions between different chromosomes, is another important reason.

Most genome-wide methods for determining chromatin structure are based on proximity ligation. Previously, inter-chromosomal interactions were considered artificial errors in Hi-C studies (Kalhor et al., 2012; Kaufmann et al., 2015; Bertero, 2021). Compared with the intrachromosomal interactions described in the previous section (e.g., TAD and compartment formation within a chromosome), the fundamental understanding of inter-chromosomal interactions remains limited (Figure 1). The nucleolus exhibits the best nuclear organization via inter-chromosomal interactions. In various eukaryotic species, including humans and mice, the nucleolus exhibits the largest nuclear organization via the coalescence of ribosomal DNA (rDNA) genes across multiple chromosomes (McStay, 2016; van Sluis et al., 2020; Mangan and McStay, 2021). However, such inter-chromosomal interactions in the nucleolus have rarely been detected in previous Hi-C studies. Yu and Lemos attempted to detect the long-range interactions among rDNAs by analyzing over 15 billion Hi-C reads from previous studies. However, the ratio of reads supporting such interactions was <1%, indicating the difficulty of recovering rDNA information from Hi-C data (Yu and Lemos, 2018). On the other hand, among the gene promoters bound by polycomb repressive complex 1 (PRC1) in mouse ES cells, an unusually strong intra- and inter-chromosomal spatial network was revealed by promoter-capture Hi-C techniques (Schoenfelder et al., 2015). Because the strongest spatial network was composed of the four *Hox* clusters, the clusters are proposed to act as central 3D nucleation points for PRC1-bound genes. This spatial network is considered to constrain genome organization and differentiation of ES cells (Joshi et al., 2015; Schoenfelder et al., 2015).

**FIGURE 2:**
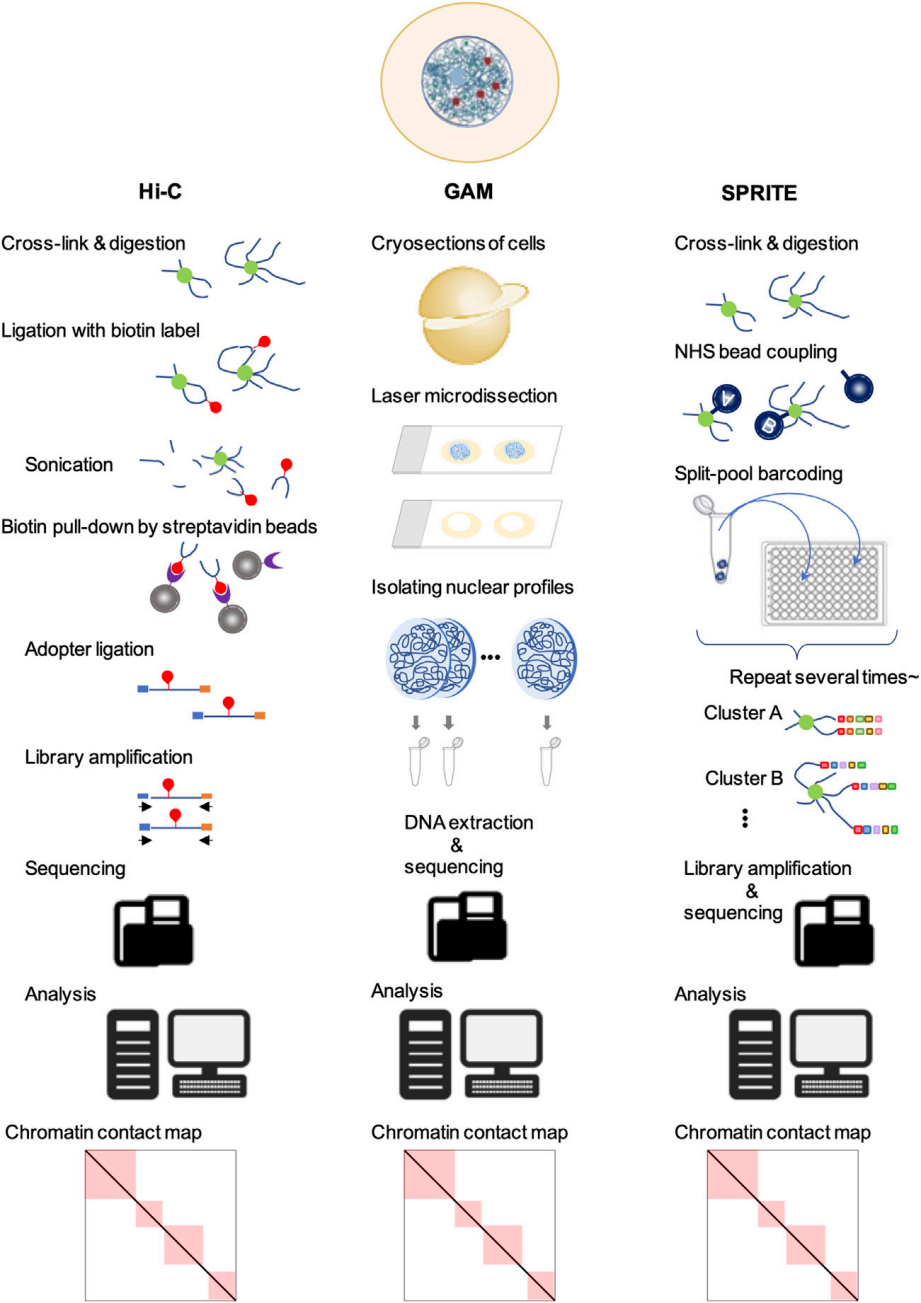
Techniques to study chromatin organization. In all of these methods, the first step is the fixation of cells for cross-linking. Next, in Hi-C, the DNA is digested, followed by different DNA regions that are in close spatial proximity and ligated with biotin (red). After end repair and adapter (blue and orange) ligation, the sequencing libraries are amplified, purified, and sequenced. Sequencing data are mapped to their genomic locations to yield genome-wide contact frequency matrices. In GAM, cells are cryosectioned into thin slices and the nuclei are isolated by laser microdissection. The DNA is extracted and sequenced. Analysis of locus co-occurrence in many sections allows proximity, including multi-way interactions, to be inferred without ligation. In SPRITE, chromatin is covalently coupled to N-hydroxysuccinimide (NHS) beads after fragmentation, followed by repeated pooling and splitting steps. The DNA fragments are barcoded sequentially. Next, the libraries are amplified and sequenced. The resulting sequencing data allow the detection of DNA fragment sequences in close proximity to each other.

In split-pool recognition of interactions by tag extension (SPRITE), the cross-linked nuclear lysate is first divided into wells of a 96-well plate, each containing a unique tag for ligation. After ligation, the samples are pooled and divided into the wells of a new 96-well plate containing different unique tags again. This process is repeated five or six times to generate more than one trillion barcodes (Quinodoz et al., 2018) (Figure 2). Therefore, SPRITE does not depend on the ligation of spatially close DNA fragments and facilitates the detection of interactions across larger distances in the genome. By applying this innovative approach to human cells, two major inter-chromosomal hubs were identified: nucleoli and nuclear speckles (Quinodoz et al., 2018). The loci clustered in the nucleoli exhibited low transcriptional activity and contained few genes. In contrast, different chromosomal loci with active gene transcription clustered in the nuclear speckles (Quinodoz et al., 2018, 2021). Furthermore, a group of genomic loci enriched in common super-enhancers is strongly involved in speckle-associated chromosomal interactions in a conserved manner across various cell types (Joo et al., 2023). This finding indicates the formation of transcriptionally active hubs in the nucleus. Similar results have been reported using another ligation-free method, genome architecture mapping (GAM) (Beagrie et al., 2017; Fiorillo et al., 2021) (Figure 2). A CRISPR-based live-cell imaging approach called 4D-CLING revealed that inter-chromosomal interactions are as common as intrachromosomal interactions (Maass et al., 2018). These new methods, not based on proximity ligation, show that inter-chromosomal interactions are common events in mammalian cells and occur at greater distances than intrachromosomal interactions.

## Inter-chromosomal interactions are involved in cell fate determination

Several studies have demonstrated the inter-chromosomal interactions between specific enhancers and target promoters. For example, within the *Tead4* promoter interactomes investigated using 4C-seq, our group identified five genomic loci that enhance *Tead4* promoter activity in a trophoblast lineage-specific manner *in vitro* (Tomikawa et al., 2020). These enhancers are located on chromosomes other than chromosome 6, where *Tead4* is located. Particularly, the enhancer located on chromosome 19 contributes to the strong trophoblast lineage-specific expression of *Tead4* at the blastocyst stage. Furthermore, inter-chromosomal enhancers promote *Pax5* gene expression in a B cell-specific manner (Fujita et al., 2017).

**FIGURE 3:**
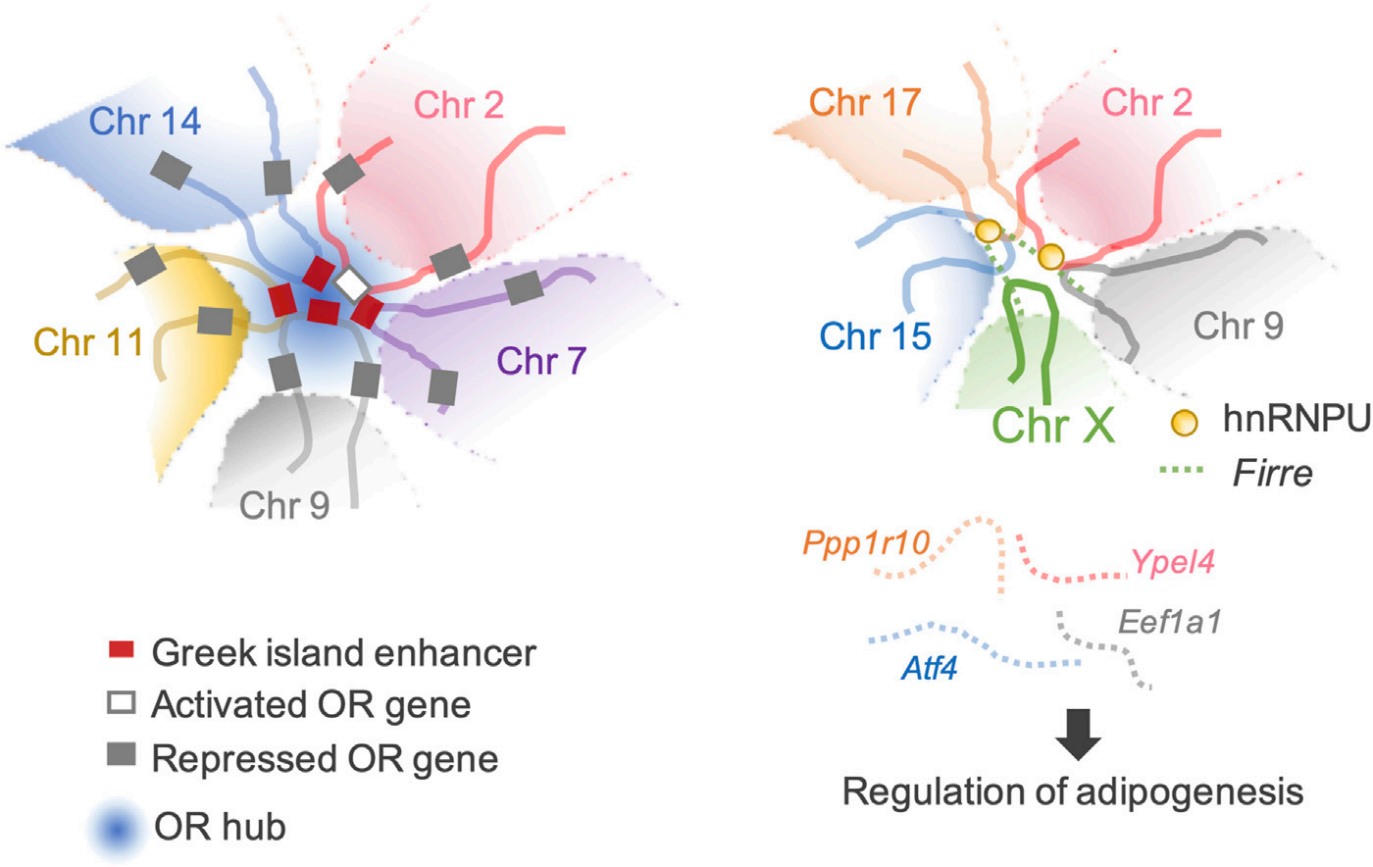
Transactivation of the target genes through inter-chromosomal genomic organization. (Left) An example of a typical *OR* hub. An *OR* gene can be activated through a concerted genomic organization that reduces the number of activating *OR*-specific enhancers from 63 available Greek Islands to 3–5 enhancer hubs. (Right) *Firre*-hnRNPU interplay mediates trans-chromosomal interactions, which promotes adipogenesis. Four genes involved in adipogenesis are organized together in spatial proximity and are co-expressed.

The most extraordinary inter-chromosomal regulator is the multi-chromosomal enhancer acting on mouse olfactory receptor (*OR*) genes (Markenscoff-Papadimitriou et al., 2014; Monahan et al., 2017). This enhancer (the Greek island enhancer) consists of 63 enhancers on 18 chromosomes. Moreover, it acts as the key machinery for removing heterochromatin marks from a single *OR* gene stochastically selected for expression among more than 1,400 *OR* genes dispersed in multiple heterochromatic gene clusters (Magklara et al., 2011; Markenscoff-Papadimitriou et al., 2014; Monahan et al., 2019) (Figure 3).

These studies indicate that interactions among different chromosomes affect gene expression. Moreover, inter-chromosomal interactions have important implications in normal cell fate determination. Although chromosome territories generally limit inter-chromosomal interactions, in exceptional cases, they allow interactions between certain genomic elements.

## Long non-coding RNAs (lncRNAs) are involved in the formation of 3D chromatin structures

In addition to DNA and histones, RNA is a major component of the nucleus (Rinn and Chang, 2012). Transcribed RNA can be divided into mRNA and ncRNA. The number of known lncRNAs of approximately 200 bp or longer is rapidly increasing because of the accumulation of RNA-seq data (Mercer et al., 2009; Wang and Chang, 2011; Derrien et al., 2012). Although the approximate number of known human protein-coding genes is 20,000, the FANTOM-5 project has identified 28,000 human lncRNAs (Hon et al., 2017). However, the functions of most lncRNAs remain unknown. Moreover, their expression levels are low, and their sequences are not highly evolutionarily conserved (Necsulea et al., 2014; de Hoon et al., 2015). Many lncRNAs exhibit spatiotemporal expression patterns (Gupta et al., 2010; Cabili et al., 2011). Well-characterized lncRNAs modulate higher-order chromatin structures (Saxena and Carninci, 2011; Marchese and Huarte, 2014).

*Xist* is the master regulator of X chromosome inactivation (XCI), a dose compensation system that balances the expression levels of X-linked genes in male and female mammals. XCI is induced by the upregulation of *Xist* expression on the future inactivated X (Xi) chromosome (Mutzel and Schulz, 2020). Female mouse pluripotent ES cells possess two active X (Xa) chromosomes (Takahashi et al., 2018). In these cells, XCI is caused by the induction of differentiation, and *Xist* accumulates in the entire Xi chromosome in cis (Herzing et al., 1997; Hoki et al., 2009). Bacher et al. (2006) analyzed differentiated female mouse ES cells. They showed that Xi and Xa segregate into separate nuclear compartments after transient co-localization of the two X chromosomes at the beginning of the inactivation process. After XCI induction, PRC1 and PRC2 are transported to the Xi chromosome, where *Xist* accumulates. Mono-ubiquitination of lysine 119 on H2A (H2Aub119) and histone 3 lysine 27 trimethylation (H3K27me3) by PRC1 and PRC2, respectively, directly block transcription (Aranda et al., 2015), induce and maintain heterochromatinization of the Xi chromosome, and suppress gene expression (Almeida et al., 2017; Masui et al., 2023). Overall, *Xist* creates a heritably silent and heterochromatic nuclear territory with a 3D structure distinct from the Xa chromosome.

*Firre*, another lncRNA that modulates local higher-order chromatin structures, is predominantly expressed on the Xa chromosome in females and localizes at an approximately 5 Mb locus, flanking its transcription site on the X chromosome. Allele-specific deletion of the *Firre* locus showed that *Firre* RNA transcribed from the Xa chromosome specifically helps anchor the PRC1 and PRC2 complexes to the Xi chromosome to maintain H3K27me3 levels and affects the nuclear localization of the Xi chromosome, which normally localizes in the vicinity of the nucleolus (Fang et al., 2020). CCCTC-binding factor (CTCF)-binding sites are located across the *Firre* locus. CTCF specifically binds to the *Firre* locus on the Xi chromosome but not to the Xa chromosome (Hacisuleyman et al., 2014; Yang et al., 2015). CTCF affects the nucleolar association of genomic loci, and the extent of CTCF binding across the *Firre* locus of the Xi chromosome is reduced by Xa chromosome-specific *Firre* deletion (Yang et al., 2015; Fang et al., 2020). These data suggest potential cooperation between *Firre* RNA and CTCF in maintaining Xi chromosome location. CTCF binding sites facilitate inter-chromosomal interactions (Botta et al., 2010). *Firre* interacts with chromosomes 2, 9, 15, and 17, which overlap with known genes including *Slc25a12*, *Ypel4*, *Eef1a1*, *Atf4*, and *Ppp1r10*. Four of these genes (*Ypel4*, *Eef1a1*, *Atf4*, and *Ppp1r10*) play regulatory roles in adipogenesis (Rubi et al., 2004; Seo et al., 2009; Lee et al., 2011; Nukitrangsan et al., 2011) (Figure 3). LncRNAs tether genes involved in similar biological processes in close proximity, facilitate spatiotemporal co-regulation, and serve as nuclear organization factors. Proper localization of *Firre* requires physical interactions with the heterogeneous nuclear ribonucleoprotein U (hnRNPU) to maintain multi-chromosomal nuclear interactions (Figure 3). Genetic deletion of *Firre* results in the loss of nuclear proximity of several inter-chromosomal loci to the *Firre* locus (Hacisuleyman et al., 2014). Similarly, many lncRNAs remain in the nucleus and interact with the chromatin to regulate their spatial structure and function (Tsai et al., 2010; Rinn and Guttman, 2014; Somarowthu et al., 2015; Zhao et al., 2020; Mattick et al., 2023).

LncRNAs are important for establishing higher-order 3D genomic structures in the nucleus. Exploring the roles of lncRNAs and their functions in dynamic assembly with other macromolecules will enhance our understanding of cellular development and evolutionary biology.

## Differentiation potential and frequency of inter-chromosomal interactions

In 2017, two groups successively reported the time-course changes of chromatin structures before and after zygotic genome activation (ZGA) examined using low-input Hi-C methods. Although their data resolution was limited, these studies showed that TAD structures are obscure in mouse zygotes and 2-cell stage embryos, which are totipotent, and are gradually established during embryonic development between 4-cell and 7.5 days post-coitum (dpc) stages (Du et al., 2017; Ke et al., 2017). Single-nucleus Hi-C analysis further revealed that TADs are formed but in an immature state in these totipotent cells (Flyamer et al., 2017; Gassler et al., 2017; Tomikawa and Miyamoto, 2021). Sperm nuclei have unique features, such as a small nuclear volume, highly condensed DNA, and loss of approximately 90% of histones. However, the TADs and A/B compartments observed in mouse sperm were similar to those observed in somatic cells. The notable difference between the 3D genome structures of sperm and somatic cells is that inter-TAD and inter-chromosomal interactions are frequently observed in sperm (Battulin et al., 2015; Ke et al., 2017; Wang et al., 2019). A comparison of the ratio of inter-chromosomal interactions among all chromosomal interactions at different stages (sperm, oocyte, zygote, 2-cell, 4-cell, 8-cell, 3.5 dpc, and 7.5 dpc) revealed that it was most abundant in sperm (∼11%) and gradually decreased during development from the zygote (9.5%) to the 8-cell stage (3%). Interestingly, it transiently increased to 6% at 3.5 dpc (blastocysts) and decreased again to 3% at 7.5 dpc (Ke et al., 2017). The occurrence of pluripotent cells in the inner cell mass may be involved in the transient increase of inter-chromosomal interactions in blastocyst. The observed dynamics of inter-chromosomal interactions suggests their potentially critical role in organizing 3D chromatin structures in the nuclei during early development. TADs are almost completely absent in early *Drosophila* embryos before ZGA and are re-established after ZGA (Hug et al., 2017). Although TADs are immature in totipotent cells in mice, ZGA occurs in these cells in a genome-wide manner (Kigami et al., 2003; Eckersley-Maslin et al., 2018). The pharmacological inhibition of ZGA did not interfere with the formation of TAD structures (Du et al., 2017; Ke et al., 2017). These observations suggest that TAD formation and ZGA are regulated independently. The abundance of inter-chromosomal interactions at the zygotes and 2-cell stages suggests the possibility that the 3D genomic architectures defined by such interactions play regulatory roles in essential developmental events, such as ZGA initiation and acquisition of totipotency. The progress of chromatin research for totipotent cells has lagged behind that for somatic cells, owing to the absence of a faithful *in vitro* model for totipotent cells and the experimental difficulty in acquiring large numbers of totipotent embryos.

## Current limitations and future challenges

To date, 3C-based methods, such as Hi-C (Fullwood et al., 2009; Lieberman-Aiden et al., 2009; Rao et al., 2014; Mumbach et al., 2016), and new methods, such as GAM (Beagrie et al., 2017) and SPRITE (Quinodoz et al., 2018), have provided insights into the genome-wide 3D spatial structure and physical proximity of DNA in the interphase nuclei. Mammalian genomes are characterized by DNA loops (Rao et al., 2014), TADs (Dixon et al., 2012; Nora et al., 2012), A/B compartments (Dekker and Mirny, 2016; Dixon et al., 2016), and other higher-order structures. These 3D structures play critical roles in the regulation of genomic functions. However, the extent to which the results obtained using the above-mentioned molecular methods are faithful to the actual 3D structure of the genome *in vivo* needs to be further carefully validated, as these methods indirectly quantify the proximity between two or more genomic loci via molecular manipulation steps and sequencing. Conversely, microscopy-based methods can directly image the target’s appearance. Microscopic techniques have advanced rapidly over the last few years (Jerkovic and Cavalli, 2021). Moreover, it is now possible to simultaneously visualize thousands of DNA loci, hundreds of different RNA molecules, and several types of protein and histone modifications, thereby enabling high-throughput chromatin structural and functional analyses in thousands of single cells (Su et al., 2020; Takei et al., 2021). These optical microscopy methods have been accompanied by the development of electron microscopy, which allows the study of genome structure at nanometer and kilobase resolutions. The application of such advanced imaging technologies provides powerful information about 3D genome organization that complements molecular techniques, such as Hi-C and SPRITE, thereby enabling the study of genome structure and function in previously unthinkable ways.
